# Central imaging based on near-infrared functional imaging technology can be useful to plan management in patients with chronic lateral ankle instability

**DOI:** 10.1186/s13018-024-04790-0

**Published:** 2024-06-18

**Authors:** Xiaoming Luo, Ben Huang, Yonglei Huang, Ming Li, Wenxin Niu, Taoli Wang

**Affiliations:** 1 Department of Rehabilitation, Xinhua Hospital, School of Medicine, Shanghai Jiaotong University, Shanghai, 200092 China; 2 Shanghai Yangzhi Rehabilitation Hospital (Shanghai Sunshine Rehabilitation Center), Tongji University School of Medicine, Shanghai, 201619 China

**Keywords:** FNIRS, Chronic lateral ankle instability, Dual task, Rehabilitation, Care

## Abstract

**Background:**

Near infrared brain functional imaging (FNIRS) has been used for the evaluation of brain functional areas, the imaging differences of central activation of cognitive-motor dual tasks between patients with chronic lateral ankle instability (CLAI) and healthy population remain unclear. This study aimed to evaluated the role of central imaging based on FNIRS technology on the plan management in patients with CLAI, to provide insights to the clinical treatment of CLAI.

**Methods:**

CLAI patients treated in our hospital from January 1, 2021 to June 31, 2022 were selected. Both CLAI patients and health controls were intervened with simple task and cognitive-motor dual task under sitting and walking conditions, and the changes of oxygenated hemoglobin concentration in bilateral prefrontal cortex (PFC), premotor cortex (PMC) and auxiliary motor area (SMA) were collected and compared.

**Results:**

A total of 23 participants were enrolled. There were significant differences in the fNIRS ΔHbO_2_ of barefoot subtractive walking PFC-R and barefoot subtractive walking SMA-R between experimental and control group (all *P* < 0.05). There was no significant difference in ΔHbO_2_ between the experimental group and the control group in other states (*P* > 0.05). There was no significant difference in ΔHbO_2_ between the experimental group and the control group in each state of the brain PMC region.

**Conclusion:**

Adaptive alterations may occur within the relevant brain functional regions of individuals with CLAI. The differential activation observed between the PFC and the SMA could represent a compensatory mechanism emerging from proprioceptive afferent disruptions following an initial ankle sprain.

## Background

Ankle sprain is one of the most common sports injuries at present, especially among the people who participate in competitive sports [1, 2], with a prevalence rate of 23%∼61% [3, 4]. The prevalence rate of external ankle sprain in the general population is also high [5]. Up to 70% of the ordinary people report having ankle injuries in their lifetime [6]. Moreover, the probability of lateral ankle re injury is as high as 80% [7]. Up to 73% of people with ankle sprains will suffer from repeated sprains [8], accompanied by a feeling of instability of the ankle [9]. The interaction of these residual mechanical injuries and sensorimotor injury symptoms promotes the development of chronic lateral ankle instability (CLAI) [10]. The instability of the ankle joint has also become a problem for more and more people [11], and the sprain injury of the lateral ankle joint has formed a higher socio-economic cost [12]. Previous studies have shown that disruptions in proprioceptive afferents of the ankle joint are considered consequences of the initial lateral malleolus sprain and may be contributory to the underlying pathogenesis of chronic ankle instability [13–15]. The poor posture and balance control ability of patients with chronic ankle instability during movement output may be related to limited joint activity and insufficient muscle strength [16, 17]. While a multitude of researchers posit that alterations in lower limb kinematics among patients with chronic ankle instability represent an adaptive protective compensation, empirical studies validating the connection between modifications in movement patterns and the presence of ankle instability remain scant [18, 19]. Therefore, motor control dysfunction in individuals with chronic ankle instability is not solely attributed to disruptions in proprioceptive afferent pathways but is also significantly associated with the neural center’s processing mechanisms and the execution of motor responses.

A dual task walking study has showed that changes in walking speed and other parameters are related to insufficient attention, and this change is particularly prominent under dual tasks with cognitive tasks [20]. Several studies have shown that dual tasks can lead to reduced motor and cognitive performance [21, 22]. Kluzik et al. [23] found that the central nervous system will change after lateral ankle injury. Currently, the literature on brain center activation in patients with chronic lateral malleolar instability during cognitive-motor dual tasks is limited. Consequently, investigating the central nervous system activation in individuals with CLAI holds substantial importance for enhancing our understanding of daily living activities, informing clinical treatment strategies, facilitating the return to work and physical exercise, and potentially providing novel insights for rehabilitation therapies. In recent years, functional near-infrared spectroscopy (fNIRS), a brain imaging technology noted for its exceptional temporal and spatial resolution, has garnered extensive interest. This innovative approach offers researchers a novel avenue for investigating brain function with greater precision and detail. This technology is capable of tracking the fluctuation in oxygenated hemoglobin (HbO_2_) concentrations, thereby providing insights into the activation of specific brain regions [24]. In the realm of brain imaging, fNIRS enables the discernment of distinct activity patterns associated with wrist movements in various directions, as revealed by the captured signal data [25]. The analysis of the recorded signals by fNIRS has revealed that each distinct direction of motion elicits a unique pattern of activation within the motor cortex in assessing cortical responses to passive wrist movements [26]. The distinctions in central nervous system activation during cognitive-motor dual tasks between individuals with CLAI and those in the healthy population have yet to be fully elucidated. Therefore, the objective of this study is to employ functional near-infrared spectroscopy (fNIRS) technology to investigate the neuroimaging alterations in central activation among patients with CLAI during dual-task conditions. The findings aim to contribute evidence towards a deeper understanding of the central mechanisms underlying the progression and therapeutic approaches for CLAI.

## Methods

### Ethics

This study was a pilot design. This study was submitted to the Ethics Committee of the institution and was approved with approval number SBKT-2022-060(2024-017). All participants were clearly informed of the purpose and process of the experiment and signed the written informed consent form.

### Participants

CLAI patients treated in our hospital from January 1, 2021 to June 31, 2022 were selected. the inclusion criteria were as following: (1) Patient had at least on history of ankle sprain and diagnosed with CLAI. The initial ankle sprain must occur 1 year before the study was included; the most recent ankle sprain must occur 3 months before inclusion in the study. (2) The injured ankle had felt “flaccid”, and/or “repeatedly sprained”, and/or “unstable” within 6 months before participating in the study. The exclusion criteria were: (1) The musculoskeletal system structure (such as bone, joint, nerve, etc.) of any lower limb had undergone surgery; (2) There was high arch varus foot caused by neuropathy; (3) There were cognitive disorders, mental illness onset or other conditions that could not cooperate with the completion of the experimental study (4) The patient was not willing to sign the informed consent form.

Additionally, alongside healthy controls for comparative analysis, the inclusion criteria for participants were designed to ensure that they had no history of foot injuries or other underlying medical conditions, and that they were willing to voluntarily participate in this study.

### Equipment

We used near-infrared brain functional imaging system (Huichuang Medical Equipment Co., Ltd., Danyang, China) for monitoring, including three wavelengths of 740 nm, 808 nm and 850 nm. The absorption peak of oxyhemoglobin was about 850 nm, the absorption peak of deoxyhemoglobin was about 740 nm, and there was an equal absorption point near 805 nm for two kinds of hemoglobin. WalkingPad S1 Pro walking machine (Xiaomi Technology Co., Ltd, Beijing, China) was used for talk test. All subjects wore tight sport shirts and shorts for gait test.

### Intervention

**Fig. 1:**
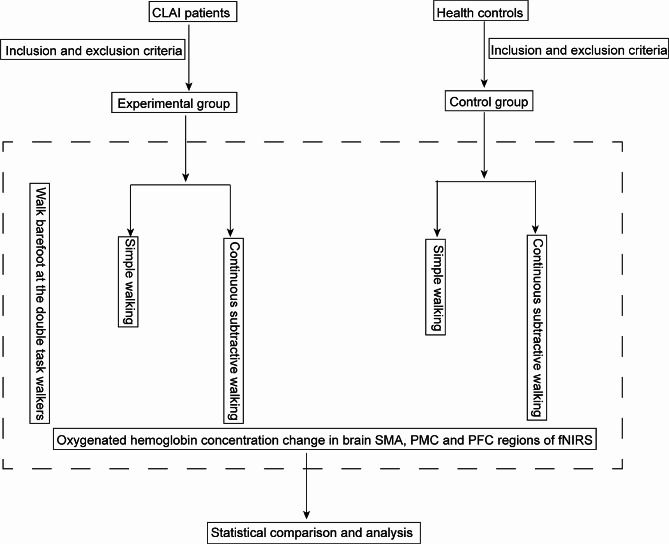
The schematic of task diagram

All participants received the cognitive task of continuously subtracting 7 while walking barefoot, and performed near-infrared brain functional imaging analysis at the same time (Fig. 1).

The cognitive-motor dual task paradigm involved performing a series of seven subtractions while walking. The task entailed selecting a random number between 200 and 250 (excluding numbers ending in 0 or 7) and instructing the participant to engage in continuous mental subtraction of seven from the chosen number during the walk. The participant was then required to verbally communicate the computed results. A total of 8 light transmitters and 10 receivers were used in this study. The near-infrared brain function of three regions including the premotor cortex (PMC), supplementary motor area (SMA), and prefrontal cortex (PFC) were observed under dual tasks.

**Fig. 2:**
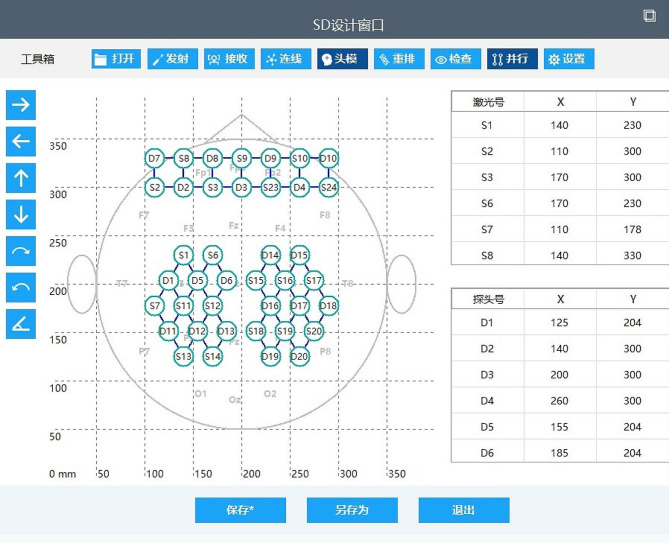
Layout of target brain regions

In the near-infrared information acquisition system, we selected and saved the target brain region of the brain model, and set the signal transmitting point and signal receiving point (adjacent two points cannot be both transmitting points or receiving points). During the configuration process, the system automatically connected each point to establish the network. When defining the transmitting and receiving points, it was crucial to ensure that the target brain area was as fully covered as possible. Once the setup was complete, a comprehensive layout was formed and saved for subsequent debugging. The target brain areas set in this study were the frontal and parietal lobes, as shown in Fig. 2.

**Fig. 3:**
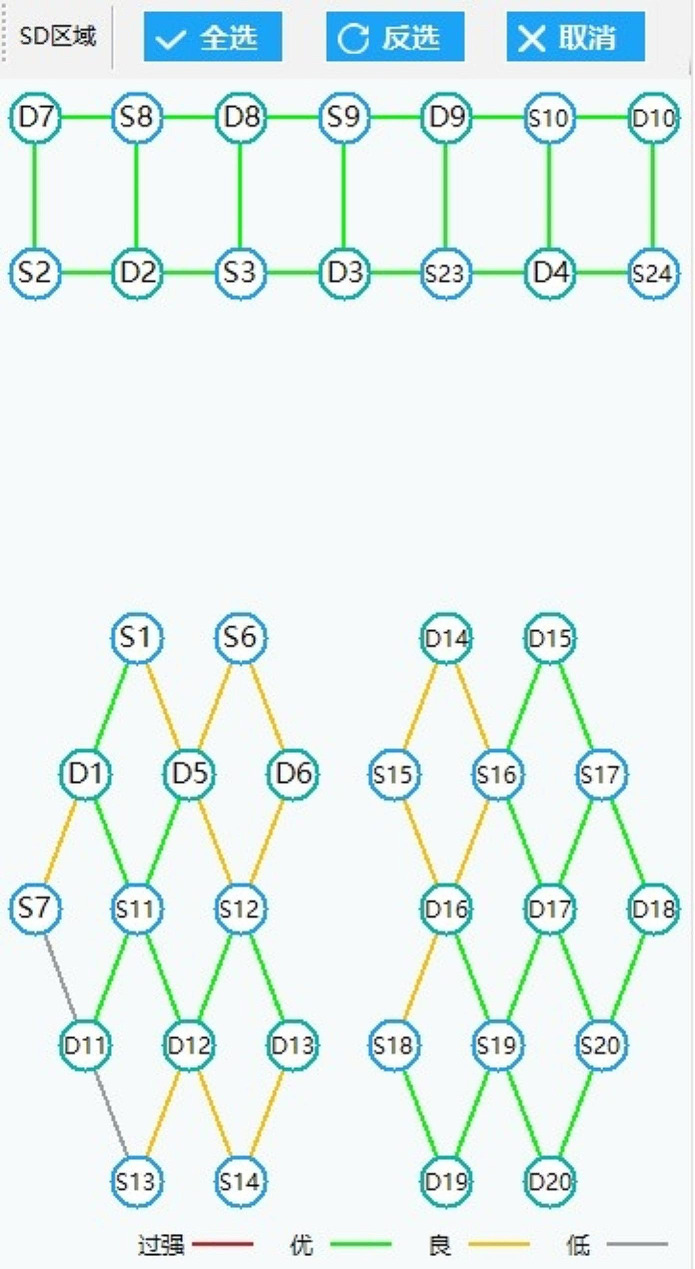
Signal debugging standard of near infrared channel

We chose the appropriate hat according to the head circumference of the subject, and installed the base of the signal probe on the hat. We determined the intersection of the connecting line between the two earlobes of the subject and the connecting line between the tip of the nose and the occipital tuberosity, which was the Cz point. According to the brain area layout, we took the Cz point as the center, determine the position of each signal receiving point and transmitting point, and installed the same color probe with the corresponding serial number on the base of the corresponding position. After the installation of all probes, the trial run software shall be debugged, and the probe to be debugged shall be determined based on the color of the line segment connecting each point (red meant that the signal was too strong, green meant that the signal was good, yellow meant that the signal was general, and gray meant that the signal was lost, Fig. 3). We repeated the above debugging process until all line segments were green or yellow and debugging was completed.

**Fig. 4:**
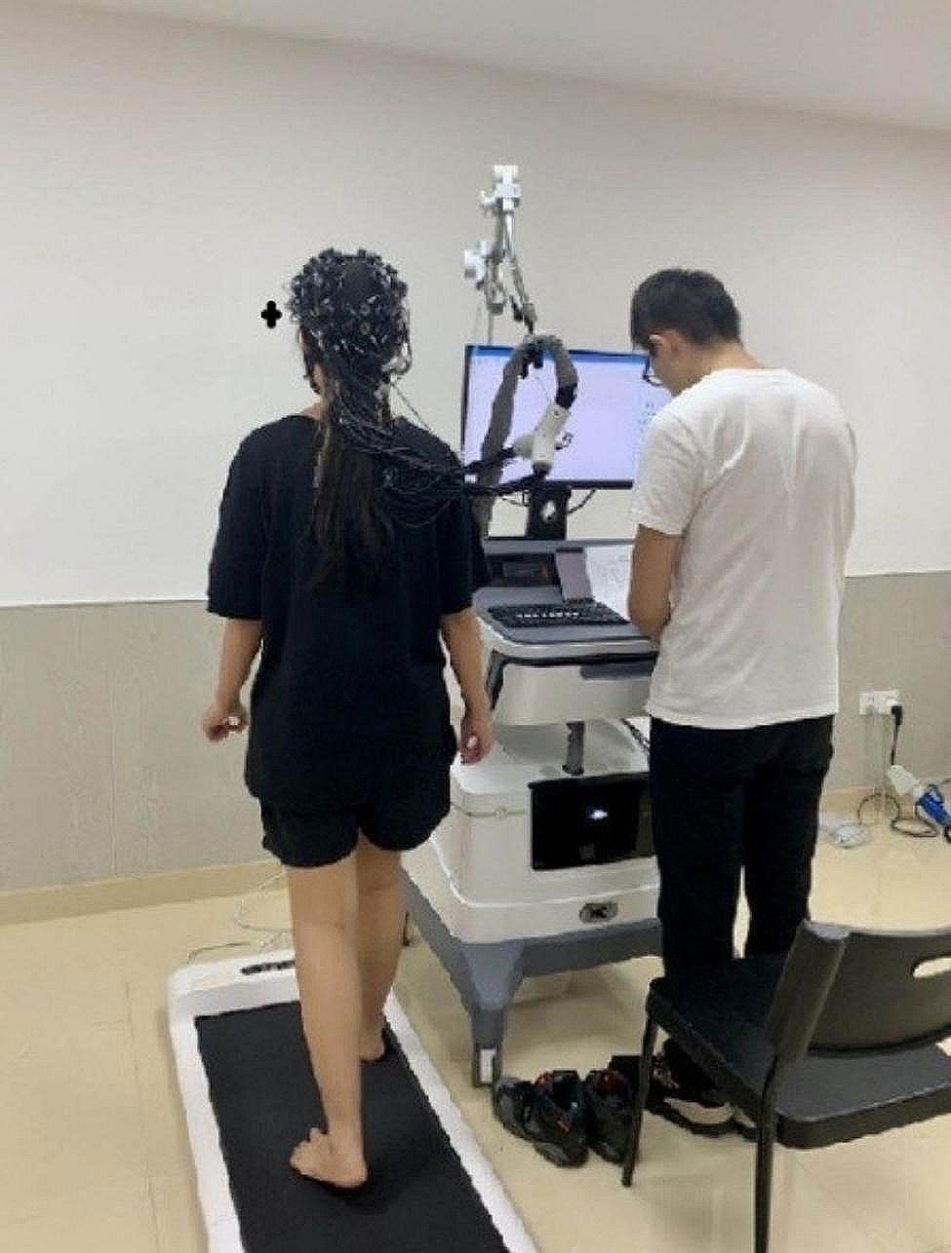
The experimental test process

We provided the participants with a thorough explanation and demonstration of the walking actions, experimental procedure, and important precautions to observe. The actual testing commenced after the subjects had completed two practice rounds. Each participant was required to undertake both single-task and dual-task interventions under conditions of seated walking and barefoot walking. Each test task was to be performed a total of three times. The walking speed of the walking machine was set to 2.5 km/h. The data shall be marked in each test state of the participant. At the beginning and end of each test state, the data shall be marked and recorded separately. Each task shall be completed 3 times, and each time shall be marked 3 times. There were 6 kinds of marks in each state, so as to collect data of 2 different tasks in 2 different states (Fig. 4).

In this study, multi-channel tissue oxygenation monitoring technology with continuous wave was used. We used the three-dimensional position measurement system (FASTRAC; Polhemus, Colchester, VT, USA) to measure the probe position. The obtained coordinates were converted to MNI coordinates and NIRS_ SPM was further projected to MNI standard brain template. Based on these data, the corresponding brain position of each channel was determined. the corresponding relationship between the test channel and PMC, SMA and PFC is shown in Table 1.

**Table 1:** Corresponding relationship between brain areas and test channels

Brain areas	Channels
PFC-L	5、6、7
PFC-R	51、52、53
SMA-L	9、26
SMA-R	33、34
PMC-L	22、25
PMC-R	37、38

PFC-L: left prefrontal lobe; PFC-R: right prefrontal lobe; PMC-L: left anterior motor cortex; PMC-R: right anterior motor cortex; SMA-L: Left brain auxiliary motor area; SMA-R: right brain auxiliary motor area

We used MATLAB 2014a and the MATLAB based fNIRS processing package HOMER2 to preprocess the original fNIRS data. In this study, we adopted the change of oxyhemoglobin relative concentration, Δ HbO_2_) signals are used as indicators of hemodynamic response, because they were more sensitive to regional cerebral blood flow than deoxyhemoglobin. We calculated the average Δ HbO_2_ value between the 5th and 25th seconds of each task.

### Statistical analysis

In this study, Shapiro Wilk method was used to test the normality of measurement data, and Levene test was used to test the homogeneity of variance. The measurement data were expressed by mean ± standard deviation, and those conforming to normal distribution were statistically analyzed by independent sample t-test. The measurement data of non-normal distribution were expressed by median, and were statistically analyzed by nonparametric rank sum test. The counting data were expressed by frequency (percentage) and statistically analyzed by chi square test. The statistical software was IBM-SPSS 21.0. The statistical significance was set as *P* < 0.05 in this study.

## Results

In accordance with the predefined inclusion and exclusion criteria, a total of 23 participants were recruited for the study, comprising 10 individuals in the experimental group and 13 in the control group. As indicated in Table 2, there was no significant difference in age, sex, height, weight, body mass index (BMI) and test foot between the two groups (*P* > 0.05). The average course of disease in the experimental group was 3.13 years.

**Table 2:** The characteristics of participant

Characteristics	Experimental group (*n* = 10)	Control group (*n* = 13)	t/F	*P*
Age(y)	23 (21, 39)	23 (22, 27)	-0.416	0.677
Gender			0.371	0.543
Male	4 (40.0%)	7 (53.8%)		
Female	6 (60.0%)	6 (46.2%)		
Height(cm)	169.50 ± 8.01	167.00 ± 10.00	0.761	0.455
Weight(kg)	65.90 ± 11.90	63.10 ± 12.80	0.567	0.576
BMI(kg/m^2^)	22.91 ± 3.80	22.47 ± 2.61	0.340	0.737
Test foot			/	0.160
Right foot	8 (80.0%)	12 (92.3%)		
Left foot	2(20.0%)	1 (7.7%)		
Duration of disease(y)	3.13 ± 2.52	/		

BMI: body mass index

The fNIRS ΔHbO_2_ in cerebral cortex during different task states are shown in Table 3. There were significant differences in the fNIRS ΔHbO_2_ of barefoot subtractive walking PFC-R and barefoot subtractive walking SMA-R between experimental and control group (all *P* < 0.05).

**Table 3:** fNIRS ΔHbO_2_ in cerebral cortex

Variables	Experimental group (*n* = 10)	Control group (*n* = 13)	t	*P*
Sitting subtractive PFC-L	0.0514 ± 0.046	0.0696 ± 0.061	-0.833	0.414
Sitting subtractive PFC-R	0.0642 ± 0.034	0.0477 ± 0.066	0.773	0.448
Sitting subtractive PMC-L	0.0733 ± 0.050	0.0861 ± 0.074	-0.500	0.622
Sitting subtractive PMC-R	0.0490 ± 0.036	0.0853 ± 0.062	-1.763	0.091
Sitting subtractive SMA-L	0.0697 ± 0.041	0.0865 ± 0.076	-0.680	0.504
Sitting subtractive SMA-R	0.0401 ± 0.028	0.0825 ± 0.063	-1.886	0.053
Barefoot walking PFC-L	0.0094 ± 0.030	0.0075 ± 0.032	0.157	0.876
Barefoot walking PFC-R	0.0035 ± 0.029	0.0025 ± 0.043	0.070	0.945
Barefoot walking PMC-L	-0.0062 ± 0.028	-0.0006 ± 0.036	-0.426	0.674
Barefoot walking PMC-R	0.0098 ± 0.042	0.0057 ± 0.049	0.221	0.827
Barefoot walking SMA-L	0.0017 ± 0.040	0.0056 ± 0.035	-0.262	0.796
Barefoot walking SMA-R	0.0124 ± 0.043	0.0105 ± 0.047	0.102	0.920
Barefoot subtractive walking PFC-L	0.0642 ± 0.075	0.0284 ± 0.050	0.755	0.158
Barefoot subtractive walking PFC-R	0.0809 ± 0.089	0.0198 ± 0.051	-2.255	0.041^*^
Barefoot subtractive walking PMC-L	0.0688 ± 0.043	0.0621 ± 0.051	0.351	0.729
Barefoot subtractive walking PMC-R	0.0273 ± 0.051	0.0348 ± 0.047	-0.381	0.707
Barefoot subtractive walking SMA-L	0.0278 ± 0.077	0.0527 ± 0.056	-0.963	0.246
Barefoot subtractive walking SMA-R	0.0193 ± 0.051	0.0546 ± 0.046	-2.146	0.048^*^

PFC-L: left prefrontal lobe; PFC-R: right prefrontal lobe; PMC-L: left anterior motor cortex; PMC-R: right anterior motor cortex; SMA-L: Left brain auxiliary motor area; SMA-R: right brain auxiliary motor area

**Fig. 5:**
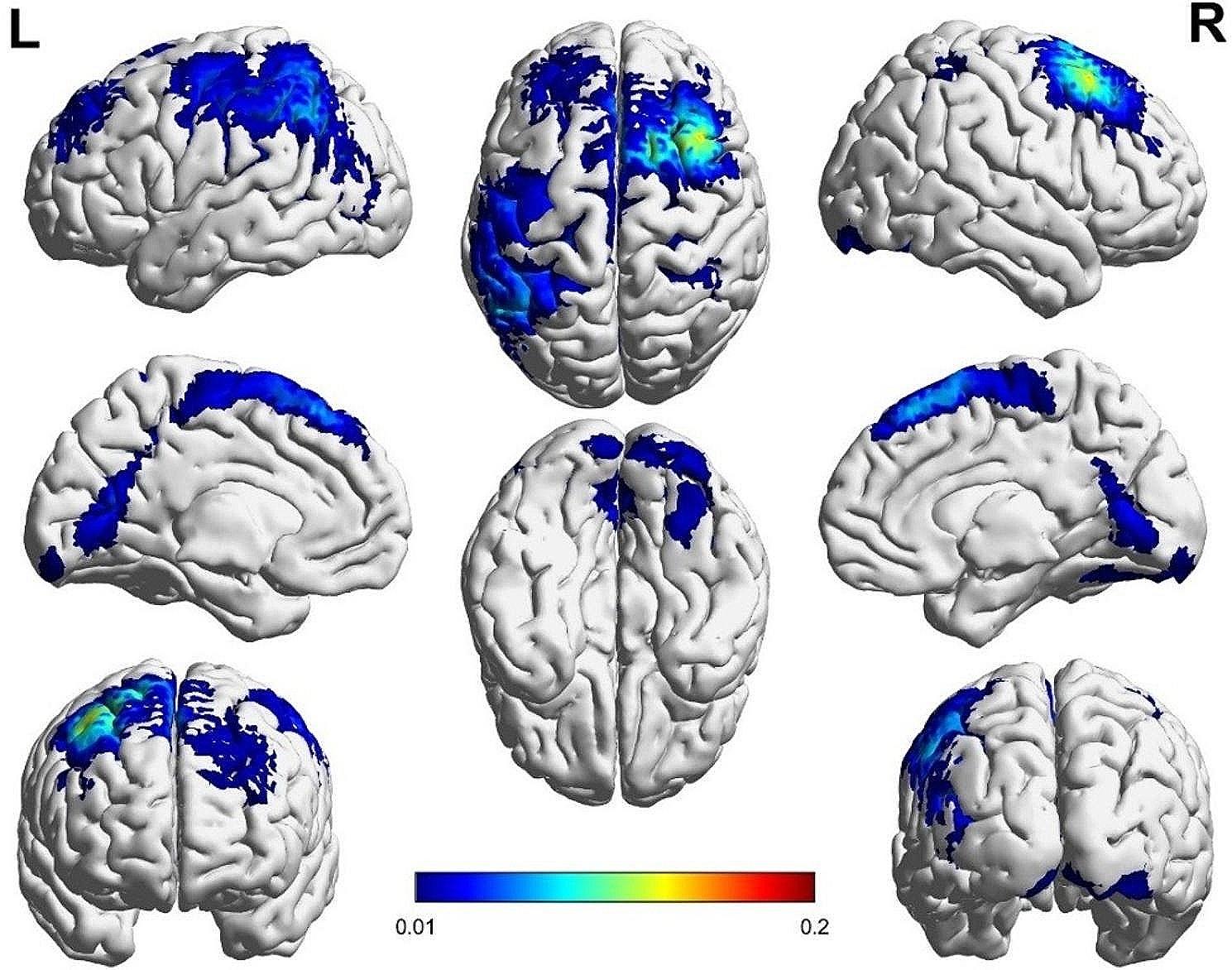
Brain activation map of barefoot subtractive walking in the control group

**Fig. 6:**
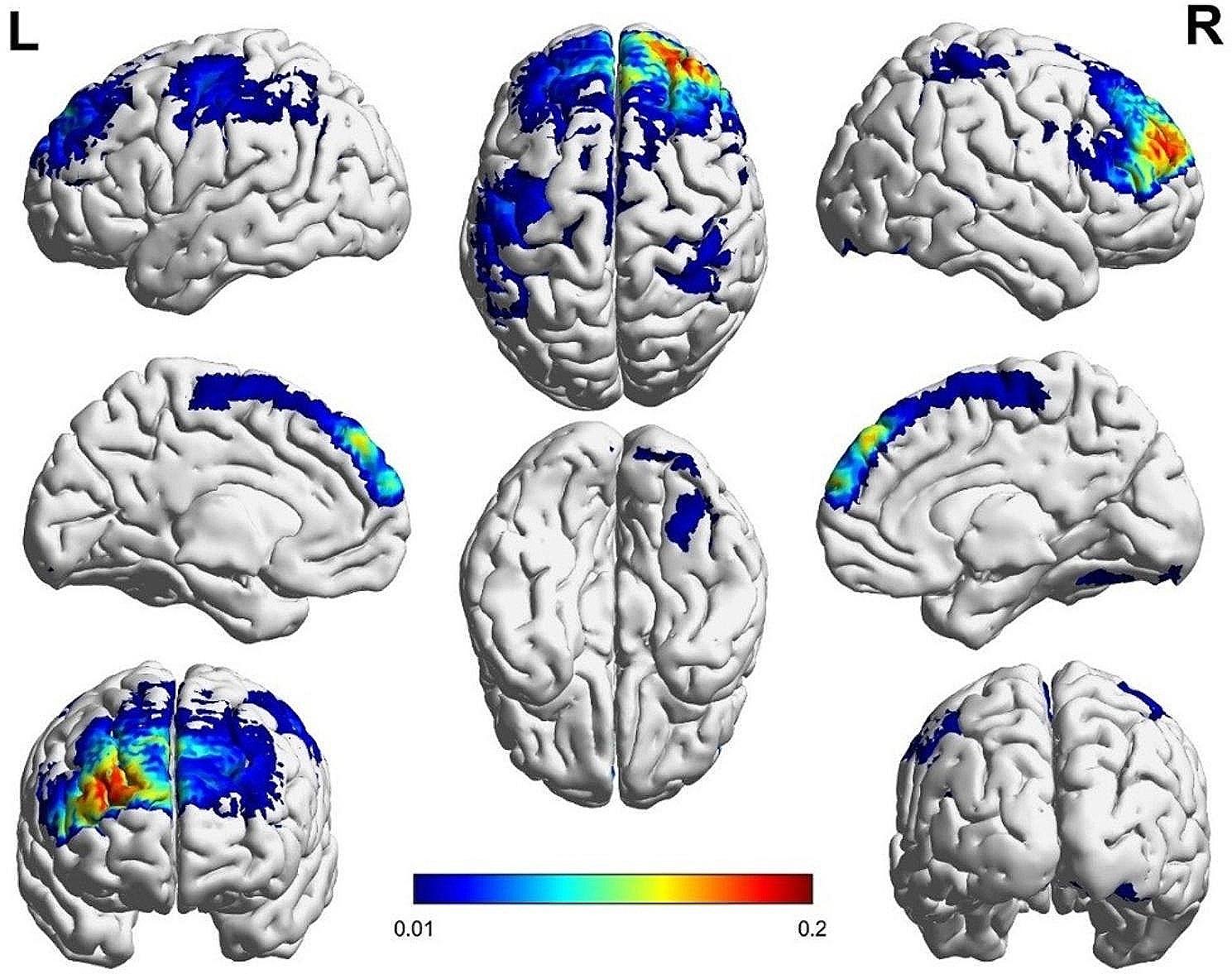
Brain activation map of barefoot subtractive walking in the experimental group

Activation of the PFC in the experimental group was markedly greater compared to the control group during the execution of dual tasks, as depicted in Figs. 5 and 6. The results showed that in the walking and sports tasks, the activation of PFC was involved in planning, organizing and performing actions, as well as adaptive adjustment to environmental changes. The SMA activation in the control group was significantly higher than that in the experimental group when performing dual tasks (Figs. 5 and 6). It showed that the activation of SMA was closely related to motion planning and execution, especially in the tasks involving complex motion sequences, which participated in the formulation of motion plans and coordinated the execution of movements.

## Discussions

In previous studies on the activation of brain functional areas during walking in healthy people, Harada et al. [27] has found that PMC, SMA and PFC are significantly activated during walking in healthy people. Miyai et al. [28] has found that the activation of bilateral auxiliary motor areas increased during walking in healthy people, and the activation of PFC, PMC and primary sensorimotor cortex (SMC) increased during walking and running. At the same time, Suzuki et al. [29] has found that PFC plays an important role in the process of adapting to the change of walking speed in healthy people. Bilateral activation of the PFC and PMC exhibits a significant increase during the acceleration phase of walking, with the SMC showing minimal variation in activation. In contrast, the medial PFC demonstrates the most pronounced changes in activation during the running phase. An fMRI study has showed an increase in PFC activation during the walk initiation phase [30] and an increase in SMA activation during the walk initiation phase [31]. Miyai et al. [32] has evaluated the changes of cerebral hemoglobin concentration in 6 stroke patients during walking. Previous study [33] has showed that the activation of frontal lobe area in healthy people decreased after walking acceleration, while the frontal lobe area in stroke patients remained active after walking acceleration. Therefore, the frontal lobe area may be a compensatory area for pace regulation.

Doi et al. [34] have found that PFC activation in the prefrontal lobe increased when the elderly performed cognitive tasks while walking, while Beurskens et al. [35] have found that prefrontal lobe activity decreased significantly when elderly people performed judgment tasks while walking, and there was no change in brain activity when walking and communicating at the same time. Task complexity seems to affect brain activity in the prefrontal lobe of young people during walking. Hill et al. [36] have found that PFC activation increased significantly only when performing more difficult cognitive tasks. At the same time, Beurskens et al. [35] have observed a significant decrease in PFC activation during visual dual tasks, while Holtzer et al. [37] have found that PFC activation increased in young people when performing oral tasks, especially when walking. Previous studies have shown that the minus 7 subtraction task (randomly selected numbers from 200 to 300 minus 7 times in a row) seems to hinder the stable activation of leg muscles in patients with CAI, while verbal memory tasks lead to more protective landing strategies [38]. Therefore, the continuous subtraction 7 subtraction task is enough to affect the functional activity performance of CAI patients. The study employs a continuous subtraction task, specifically subtracting seven repeatedly, as an oral component that mandates participants to announce their calculation outcomes in an ongoing manner. This approach enhances the task’s complexity and facilitates a more distinct observation of brain functional area activation changes in patients with CLAI.

Previous studies [39, 40] have shown that subcortical and subcortical regions are involved in stable bipedal walking, which requires specific motor networks in the brain. The direct pathway guides the movement through the M1 area, cerebellum and spinal cord, while the indirect pathway regulates the movement through the prefrontal cortex, auxiliary motor area and basal ganglia [41]. In addition, the prefrontal cortex is important for top-down regulation [42] and promoting subcortical motor pathway connections [43]. Some activation of the prefrontal cortex and anterior cingulate cortex when walking with specific goals, more complex walking tasks, and dual-task walking. However, the prefrontal cortex is also more active during fast walking [44]. The increase in PFC activity may need to distinguish between related stimuli and irrelevant stimuli. SMA involves non-speed control and exercise plan execution [45], which may be a necessary factor to ensure posture and motor stability during high-speed movement.

Reduced activity in specific brain regions may be a compensatory strategy for older individuals to ensure the activity of a wide range of cortical networks needed to successfully solve tasks [46–48]. The overactivity in specific brain regions of patients with nervous system also reflects the compensation strategy described by Stern [49]. The co-activation of multiple sensory areas in the elderly may be a compensatory strategy for peripheral sensory defects. The elderly are more dependent on the co-activation of multiple sensory areas, while the young show a task-oriented activation model [50]. The increase in prefrontal cortex and SMA activity in the elderly [51] also reflects a compensatory strategy. Kluzik et al. [23] have shown that after chronic ankle instability and other musculoskeletal system injuries, the central processing system will also have adaptive changes, in which the reduced functional areas will be dominated by other functional areas, which may be one of the reasons for the kinematic changes of the lower extremities. There is no difference in the change of HbO_2_ in cerebral cortex between CAI and healthy subjects, but the variability of HbO_2_ concentration in patients with CAI is greater than that in healthy subjects, indicating that the strategy of cortical activation may be changed to maintain limb balance [52].

Changes in exercise preparation or feedforward exercise plans, and changes in SMA activities indicate that this feedforward control has been affected. Therefore, this change in cortical activation may be an adaptive change that plays a role in successfully coordinating dynamic tasks [53, 54]. Different from the results of previous studies, we have found that there are significant differences in the activity of right PFC and right SMA between the experimental group and the control group during dual tasks. During the dual-task conditions, the experimental group exhibited significantly heightened activity in the right PFC compared to the control group. Conversely, the activation of the SMA on the right side was notably reduced in the experimental group relative to the control group. These findings suggest that adaptive alterations may be occurring within the brain’s functional regions in patients with CLAI. Patients with CLAI need higher attention control and motor control executive function in PFC area. At the same time, the degree of activation of the SMA area, which dominates non-speed control and exercise planning, may be one of the causes of postural control and motor stability disorders, and indirectly lead to the occurrence and development of chronic ankle instability [55, 56].

The fNIRS technology utilized in this study represents a novel method for monitoring brain function, which has been extensively applied in the diagnosis, treatment, and research of cerebral functions, as well as in the monitoring of pain processes [57–60]. There are still some limitations in this study. First, the sample size used in this study is small. Secondly, the cognitive-motor dual task used in this study is the oral subtraction task, and the dual tasks such as image discrimination and continuous conversation are not used for comparison. In addition, our study takes the change of HbO_2_ concentration as the target parameter, excluding indicators such as HbR and HbT, and does not include parameters such as the slope and peak value of the hemoglobin curve. Therefore, in the future studies, it is necessary to expand the sample size and extract more characteristic parameters to obtain more comprehensive research results.

## Conclusions

The results of this study have showed that the PFC of CLAI patients is more active than that of healthy controls, while the activation of SMA area decreases significantly. The increase of PFC activation may be due to the fact that CLAI patients need more attention allocation and motor control functions when performing dual tasks. At the same time, the decrease of SMA activation indicates that the ability of posture control and exercise planning is decreased in CLAI patients, which suggests that adaptive changes may have taken place in the brain functional areas of CLAI patients. Previous studies have also shown that there will be adaptive changes in the central processing system of patients with CAI, and limited brain resources make individuals form compensatory strategies [61, 62]. The disparity in activation between the PFC and the SMA may not solely represent a compensatory strategy emerging from proprioceptive afferent disruptions following an initial ankle sprain. It could also contribute to a diminished capacity for posture control and motor stability, potentially exacerbating the progression towards CLAI. To elucidate the relationship between the activation changes in brain functional areas involved in sensory integration, motor execution, and posture control, and the development of CLAI, further studies with larger participant sample size are warranted. Additionally, investigating the connection between the excitability of sensory and motor conduction tracts of the spinal cord and CLAI could provide valuable insights.

## Data Availability

All data generated or analyzed during this study are included in this published article.

## Abbreviations

CLAI: chronic lateral ankle instability
fNIRS: near infrared brain functional imaging
PFC: bilateral prefrontal cortex
PMC: premotor cortex
SMA: auxiliary motor area
HbO_2_: oxygenated hemoglobin
